# Perceptions on the use of pricing strategies to stimulate healthy eating among residents of deprived neighbourhoods: a focus group study

**DOI:** 10.1186/1479-5868-7-44

**Published:** 2010-05-19

**Authors:** Wilma E Waterlander, Anika de Mul, Albertine J Schuit, Jacob C Seidell, Ingrid HM Steenhuis

**Affiliations:** 1 Department of Health Sciences and the EMGO Institute for Health and Care Research, Faculty of Earth and Life Sciences, VU University Amsterdam. De Boelelaan 1085, 1081 HV Amsterdam, the Netherlands; 2 National Institute for Public Health and the Environment. Postbus 1, 3720 BA Bilthoven, the Netherlands

## Abstract

**Background:**

Pricing strategies are mentioned frequently as a potentially effective tool to stimulate healthy eating, mainly for consumers with a low socio-economic status. Still, it is not known how these consumers perceive pricing strategies, which pricing strategies are favoured and what contextual factors are important in achieving the anticipated effects.

**Methods:**

We conducted seven focus groups among 59 residents of deprived neighbourhoods in two large Dutch cities. The focus group topics were based on insights from Rogers' Diffusion of Innovations Theory and consisted of four parts: 1) discussion on factors in food selection; 2) attitudes and perceptions towards food prices; 3) thinking up pricing strategies; 4) attitudes and perceptions regarding nine pricing strategies that were nominated by experts in a former Delphi Study. Analyses were conducted with Atlas.ti 5.2 computer software, using the framework approach.

**Results:**

Qualitative analyses revealed that this group of consumers consider price to be a core factor in food choice and that they experience financial barriers against buying certain foods. Price was also experienced as a proficient tool to stimulate healthier food choices. Yet, consumers indicated that significant effects could only be achieved by combining price with information and promotion techniques. In general, pricing strategies focusing on encouraging healthy eating were valued to be more helpful than pricing strategies which focused on discouraging unhealthy eating. Suggested high reward strategies were: reducing the price of healthier options of comparable products (e.g., whole meal bread) compared to unhealthier options (e.g., white bread); providing a healthy food discount card for low-income groups; and combining price discounts on healthier foods with other marketing techniques such as displaying cheap and healthy foods at the cash desk.

**Conclusion:**

This focus group study provides important new insights regarding the use of pricing strategies to stimulate healthy eating. The observed perceptions and attitudes of residents of deprived neighbourhoods can be integrated into future experimental studies and be used to reveal if and how pricing strategies are effective in stimulating healthy eating.

## Introduction

The prevalence of overweight and obesity is rising worldwide, with the largest burden among lower socio-economic groups [1-3]. These figures are worrying since overweight and obesity are related to several chronic diseases, including type 2 diabetes mellitus, different types of cancer, and cardio vascular diseases [4]. Nowadays, pricing strategies are seen as promising strategies in reducing or halting this public health problem. Arguments for introducing pricing strategies on food can be found in different areas [5,6].

The first argument departs from marketing science; sales promotions form an important part of the marketing mix, and are used widely as an incentive to buy certain products [7,8]. Based on this, pricing strategies may also be successful in stimulating healthy eating. Another argument is based on neoclassical economic theory. This theory argues that consumers' choices are constrained by their available resources, and that the number of purchased products is a function of income, price and taste [9]. Since there exists a large body of evidence showing that nutrient-rich, low-energy-dense foods (i.e., fruits and vegetables) are generally relatively more expensive than high-energy-dense, fat and sugar rich foods [10,11], consumers with a low socio-economical status (SES) may perceive financial barriers to healthy eating. In addition, it is suggested that in the current market-driven economy, fruit and vegetables are promoted less than more lucrative, highly processed foods containing higher levels of fats and sugars [12,13]. Finally, there are arguments that are based on consumers' motives for food choice. Studies found that besides taste and quality, price is the main factor in food choice, and that price is especially significant for low SES consumers [14-16]. Furthermore, a study conducted among low-income consumers revealed a negative perception towards the price of fruit and vegetables in particular [17]. Next to the listed arguments, there is some evidence regarding the price elasticity of food. Experiments in controlled settings showed that price reductions are effective in changing food purchases [18] and studies on fiscal incentives revealed that taxes and subsidies on food may be effective in stimulating healthy eating [19-22].

The above arguments imply that pricing strategies are a convenient tool in stimulating healthy eating, especially among low SES consumers. In a recently conducted Delphi study, we examined expert viewpoints on what kind of pricing strategies are most feasible and effective. The expert panel agreed on the potential success of offering small presents, providing price-cuts on healthy foods and discounting healthier foods more frequently [23]. Although this is promising, we do not know how consumers perceive pricing strategies and what contextual factors are important in achieving in the anticipated effects. Before pricing strategies can be implemented successfully it is important to gain insight into these processes. Therefore, the aim of this study is to reveal consumers' attitudes and perceptions towards pricing strategies, and to gain insight into surrounding contextual variables that should be taken into account when implementing these strategies.

## Methods

We conducted seven focus groups in two cities in the Netherlands. Owing to the interactive, groups process, focus groups are effective in gathering new insights into an unexplored field [24]. The method and procedures of this study were approved by a local Medical Ethical Committee.

### Participants

People were approached through community centres in deprived neighbourhoods of two large Dutch cities, defined by postal code area status scores of the Dutch Social and Cultural Planning Office (SCP). The SCP uses average income, number of households with a low income, number of unemployed people and number of people with a relatively low educational level as indicators for the status scores [25]. Participants were recruited using purposive sampling methods by engaging with key persons from community centres [24]. Community centres were chosen because they are closely linked with social work and welfare services. Inclusion criteria were that the participants had to be aged 18 years or older and living independently. We first conducted four focus groups among native Dutch participants, second, we conducted additional focus groups among Turkish and Moroccan immigrants, the two largest migrant groups in the Netherlands [26]. This extra emphasis was included since a large proportion of lower income groups are from immigrant populations, which may have different dietary habits compared to the original Dutch population and should, therefore, be included to achieve a complete overview of attitudes of low-income consumers. All participants received a small monetary reward for their participation.

### Description of the focus groups

The focus groups were carried out according to regular procedures [24]. All focus groups were audio-recorded and conducted with two researchers; one interviewer and one minute's secretary taking field notes and asking extended questions if necessary. Throughout the sessions an effort was made to involve all participants, and they were encouraged to express their opinions. The participants were not aware of the actual interests of the study and not familiar with the research team. The focus groups followed a semi-structured format and took approximately 60 to 90 minutes.

### Focus group topics

The purpose of the focus groups was to gain insight into consumers' viewpoints regarding pricing strategies and into important contextual variables. In order to accomplish this, all focus groups consisted of four main parts, including: 1) discussion on attitudes and perceptions regarding aspects of food selection; 2) attitudes and perceptions towards food prices; 3) thinking up pricing strategies; 4) attitudes and perceptions regarding nine promising pricing strategies that were derived from a previously conducted Delphi Study [23]. In this Delphi study a group of experts representing a) Academic research, b) Food processing, Industry, Retail, Agriculture, and c) Policymakers, Public interest, and Non-Governmental and Consumer organizations, discussed feasible and effective pricing strategies in three subsequent rounds of talks. After the second round, the experts indentified fifteen potentially effective strategies. These strategies were redefined into nine comprehensive pricing strategies (some strategies were combined), which were proposed to the consumers in the focus groups. This tactic was used to gain insight into consumer perceptions concerning pricing strategies that had been identified as promising by the group of experts, and to find out whether the expert and consumer opinions overlap. With regard to these nine pricing strategies, we asked specific questions based on theoretical insights from the Diffusion of Innovations Theory (Rogers, 1983) [27]. Rogers distinguishes several attributes that are important in the uptake of innovations. Five of these attributes were relevant to this study and were processed into the topic list: relative advantage; compatibility; complexity; impact on social relations; and the communicability of the pricing strategies. An outline of the topic list is presented in Table 1. This table also includes the effectiveness scores of the nine pricing strategies as given by the experts in the Delphi study.

**Table 1:** Overview of the focus group topic list.

	Focus group section	Focus group topics	**Delphi effectiveness score (median)**^a^
1	Attitudes and perceptions on aspects of food selection	- Aspects of food selection - Reasons for healthy/unhealthy eating - Capability of eating healthy	

2	Attitudes and perceptions towards food prices	- Attitudes and perceptions towards prices of healthy and unhealthy food - Importance of price in food selection	

3	Thinking up pricing strategies	- Opinion about pricing strategies as tool to stimulate healthy eating - Thinking up pricing strategies	

4	Attitude and perception regarding nine pricing strategies from Delphi Study	**1**. Prohibition of discounts on unhealthy food items: *a*. *Overall opinion; b. Which products; c. Positive/negative aspects*; *d. Usefulness; e. Need for consumers; f. Leads to different food choices? g. Is it patronizing? h. Compatibility*	3
		**2**. Allowance for low-income groups designed to purchase healthy food: *a. Overall opinion; b. Which products; c. Positive/negative aspects; d. Magnitude of the allowance; e. Usefulness; f. Need for consumers; g. Leads to different food choices? h. Is it patronizing? i. Complexity/communicability*.	2
		**3**. Healthy food options being on offer more frequently: *a. Overall opinion; b. Which products; c. Positive/negative aspects; d. Magnitude of the offers; e. Usefulness; f. Need for consumers; g. Leads to different food choices? h. Is it patronizing? i. Complexity/communicability*.	5
		**4**. Healthy food items discount card exclusively for low-income groups: *a. Overall opinion; b. Which products; c. Positive/negative aspects; d. Discount magnitude; e. Usefulness; f. Need for consumers; g. Leads to different food choices? h. Is it patronizing? i. Complexity/communicability*.	3
		**5**. Offering small presents, extras or saving stamps with healthy food items: *a*. *Overall opinion; b Which products; c. Positive/negative aspects; d. Usefulness; e. Need for consumers; f. Leads to different food choices? g. Is it patronizing? h. Compatibility? i. Complexity/communicability*	4
		**6**. Making healthy food items cheaper and unhealthy food items more expensive: *a. Overall opinion; b. Which products; c. Positive/negative aspects; d. Magnitude of the price differences; e. Usefulness; f. Need for consumers; g. Leads to different food choices? h. Is it patronizing? i. Complexity/communicability*	4
		**7**. Subsidizing healthy foods: *a. Overall opinion; b. Which products; c. Positive/negative aspects; d. magnitude of subsidy; e. Usefulness; f. Need for consumers; g. Leads to different food choices? h. Is it patronizing? i. Complexity/communicability*.	4
		**8**. Tax increase on unhealthy food items: *a. Overall opinion; b Which products; c. Positive/negative aspects; d. Magnitude of the tax; e. Usefulness; f. Need for consumers; g. Leads to different food choices? h. Is it patronizing? i. Complexity/communicability*.	3
		**9**. Insurance premium cutback when a healthy diet is comprised: *a. Overall opinion; b. When allocated; c. Positive/negative aspects; d. Magnitude of premium cutback; e. Usefulness; f. Need for consumers; g. Leads to different food choices? h. Is it patronizing? i. Complexity/communicability*.	3

^a ^In the Delphi study, the experts (n = 44) judged the feasibility of the pricing strategies on a 7-point Likert scale. The scores in the Table present the median [23]

### Data analysis

The focus groups were transcribed verbatim. The transcripts were coded and analysed with Atlas.ti 5.2 computer software, using the framework approach. This approach was developed specifically for policy-relevant, qualitative research. The approach is inductive in the sense that it reflects the original focus groups; however, it starts deductively from predetermined theoretical insights and objectives [28]. The analytic procedure in our study was comparable to a previous focus group study conducted on pricing strategies to stimulate physical activity, and was based on the following steps: (1) familiarization with the data; (2) highlighting quotations in the transcripts; (3) assigning codes to the quotations; (4) reassigning the codes into larger families (5); and rearranging the families into the thematic framework [29]. The coding and reassignment of the data was conducted independently by two researchers (AM and WW). Differences in reading and explanations were minimal, and consensus was easily achieved.

### Data saturation

Focus groups were conducted until data saturation was reached. This point was defined based on previous methods by Guest et al. (2006) by counting new quotation codes per focus group transcript [30]. From the analytic procedure, a total 100 of different codes were addressed to the quotations. The majority of these codes were defined and assigned to the quotations in the first group of focus groups. After the sixth focus group, 94% of the codes were assigned and, at the seventh focus group, 96% of all codes were allocated. At this point, we assumed that an additional focus group would not provide significant new information.

## Results

In total 59 people (30 male; 29 female) participated in 7 focus groups. The number of participants ranged from 6 to 12 per focus group. 22 Participants were low educated (i.e, lower general secondary education or below), and 26 had an income below standard (Table 2). The results described below are written down based on the participants' pronouncements and must not be viewed as factually defined.

**Table 2:** Participant characteristics.

	**n**	
	
Sex (n = 59)		
- Male	30	
- Female	29	
	
Ethnicity (n = 59)		
- Native Dutch (4 focus groups)	27	
- Turkish immigrant (2 focus groups)	21	
- Moroccan immigrant (1 focus group)	11	
	**mean**	**range**
Age (n = 47)	46.2	19 - 73
Expenditures on groceries per week (n = 34)	€ 91.30	€ 10 - 150
Household size (n = 44)	3.2	1 - 6
	
	**n**	
	
Education level (n = 48)		
- No education	1	
- Primary school	11	
- Lower general secondary education	10	
- Higher secondary education/pre-university education	11	
- Intermediate vocational education	2	
- Higher vocational education/University	13	
Net annual income (n = 41) ^a, b^		
- Less than € 10.000	5	
- € 10.000-15.000	10	
- € 15.000-20.000	11	
- € 20.000-30.000	3	
- € 30.000-40.000	7	
- € 40.000+	5	
Work status (n = 41)		
- Employed	16	
- (partially) Unfit for work	6	
- Unemployed/social security	12	
- Retired	5	
- Other	2	

^a^. The standard net annual income in the Netherlands (in 2008) was € 19.116 [47]

^b^. National statistics reveal that (in 2007) 52.7% of the Dutch households had an income above standard [48]

### 1. Attitudes and perceptions regarding aspects of food selection

Participants named various determinants that they considered important in food selection. Aspects named more than once were: taste; calorific content; freshness; convenience (e.g., preparation time or availability); cultural or religious regimens (named by the Turkish and Moroccan participants); and social influence (e.g., preference of their children). Aspects named most frequently were: price; finance management; and whether foods were organic or not. An illustrative claim was:

Woman, group 5: *'You cannot always choose the healthy food of your preference. I know you need the vitamins, but because of price things turn out differently.'*

A majority of the respondents specified their own eating pattern as quite healthy, but acknowledged unhealthy eating as being common. Grounds named for consuming unhealthier foods were: difficulties in distinguishing between healthy and unhealthy foods; convenience; limited time; taste; availability; temptation; social influence (mainly from children); and price. Furthermore, the Turkish and Moroccan participants stated that unhealthy eating habits are part of their culture (e.g., offering large meals to guests and a tendency to add sugar to most foods). In general, healthy eating was viewed as being a hard job.

### 2. Attitudes and perceptions towards food prices

Appendix 1 provides an overview of the main perceptions regarding food prices recorded in the focus groups. A widely held view was that food, in general, is expensive and that food has become more expensive over the past few years. A majority argued that in particular fruit, vegetables, meat, and dairy products are expensive. Numerous participants were economizing on the purchase of these food items. Besides being cheaper, unhealthy food was considered to be tastier, easier to obtain and easier to prepare than healthy food. It was stated that:

Woman, group 4: *'Convenience food is a lot cheaper. It is cheaper buying crisps than buying fruit.'*

Man, group 1: *'There are a lot of places selling unhealthy food. There is no healthy snack bar. Unhealthy food is just around the corner... it is easy.'*

Not the entire group shared this opinion. Some participants stated that healthier foods can be selected for a fair price if seasonal foods or discounted items are taken into account. One opinion was that price is not a valid concern in affluent countries such as the Netherlands. None of the participants stated that price is not at all important and everyone looked at prices when buying food. This was also the case with respect to organic foods. Nearly all participants were in favour of selecting these foods for health, animal welfare, and food quality reasons.

Woman, group 4: *'I know chicken is healthy, but when you can buy a kilogram of chicken for such a low price, I wonder how healthy this chicken really is.'*

Still, none of the respondents purchased exclusively organic foods; they clarified that this was unaffordable.

Man, group 6: *'A regular person, with an average income, cannot afford to eat organic foods three or four times a week.'*

Participants viewed price as an important factor in food selection. When asked in an open-ended question (what do you consider to be important in food selection?) both price and finance management were named most frequently. Also price was viewed as a barrier to buying certain foods:

Woman, group 7: *'I don't base my food choice on what I would like to eat; it is determined by the price.'*

Woman, group 5: *'Food selection is really dependent on the price. A good buy, that is also healthy, is really difficult to obtain, sometimes even too difficult. You won't succeed.'*

### 3. Thinking up pricing strategies

We asked participants to think up pricing strategies that they viewed as being promising. Strategies that were mentioned included: introducing an exclusive food court for low-income families; providing two healthy products for the price of one; and price temptation techniques (e.g., signs; advertisements). Also lowering the prices of healthy foods while raising the prices of unhealthy food items were mentioned more than once.

Woman, group 5: *'Healthier foods should be made cheaper and unhealthier foods should be made more expensive. Also we should cut down on advertising of unhealthy foods and spend the saved money on healthy foods. Healthy foods should draw more attention, it is about temptation.'*

Numerous participants stated that consumers can be tempted to buy certain products by combining price and promotion strategies, for example, offering small presents with healthy foods. It was argued that mainly children are tempted to choose unhealthy foods by fancy packaging and appealing gifts. Also the focus groups agreed that when discounts are provided on healthier foods, this should be supported by widespread commercials and advertisements (e.g., this product is healthy and on offer). On the other hand, advertising of unhealthy foods could be restricted. Other strategies pointed out were to display cheap and healthy foods at the cash desk and ensure that healthy products are more eye-catching in the supermarket. Finally, there was a consensus that there is a need for clear-cut information in relation to the healthiness, ingredients and production processes of food. Healthy products were perceived as being too expensive, but also their genuine healthiness was doubted. Conflicting information prevented several participants from switching to a healthier diet. Therefore, it was argued that pricing strategies should be introduced along with fair education about the healthiness of food.

Woman, group 7: *'How can one know what healthy food is? You have to know how the food is produced... all the additives that foods contain...'*

### 4. Attitudes and perceptions regarding nine pricing strategies retrieved from a Delphi study

In general, pricing strategies focussing on encouraging healthy eating were considered to be more constructive than pricing strategies focussing on discouraging unhealthy eating. Below we will present the main responses towards the discussed nine pricing strategies. An outline is presented in Table 3.

**Table 3:** Responses towards the nine specifically asked pricing strategies.

	Pricing strategy	Positive (++)	Negative (--)
1	Tax increase on unhealthy food items	- unhealthy food may become less attractive	- may result in opposite effects - patronizing - is not effective, food remains attractive - is regressive

2	Subsidizing healthy foods	- motivating - encouraging to buy more healthy food - direct effect - applies to whole population	- someone has to pay for the allowances

3	Allowance for low-income groups designed to purchase healthy food	- extra money may result in buying more healthy food	- extra money may not be spent on healthy foods - indirect effect - restricted to low-income consumers

4	Insurance premium cutback when a healthy diet is comprised	- motivating - encouraging to buy more healthy food	- difficult to implement - unverifiable - indirect effect

5	Healthy food options being on offer more frequently	- motivating - stimulating to buy more healthy food - direct effect	- saved money may not be spend on healthy foods

6	Prohibition of discounts on unhealthy food items	- fair (especially involving children) - discouraging	- patronizing - difficult to implement

7	Offering small presents, extras or saving stamps with healthy food items	- motivating - encouraging to buy more healthy food (especially for children)	- none listed

8	Making healthy food items cheaper and unhealthy food items more expensive	- fair - encouraging to buy more healthy food - more effective than only providing discounts on healthy foods	- difficult to implement

9	Healthy food items discount card exclusively for low-income groups	- fair - encouraging to buy more healthy food - discount cards are heavily used and effective	- indirectly, people have to pay for such allowances - restricted to low-income consumers

#### I. Prohibition of discounts or special offers on unhealthy foods

The common response towards this measure was disapproval; the majority judged the measure as too excessive. One participant was strongly in favour of this tactic, stating that consumers should be protected. In one focus group, it was noted that such measures would be appropriate in places mostly visited by children such as schools and sports canteens.

#### II. Providing an allowance for low-income groups designed to purchase healthy food

There were mixed responses towards this pricing strategy. One section of the respondents believed that receiving extra money would lead to the purchase of higher amounts of healthy foods; another section believed that people would spend the extra money on other items. Participants had mixed opinions about whether such an allowance should only apply to low-income groups. It was believed that high-income consumers also should be stimulated to purchase more healthy food.

#### III. Healthy foods being on offer more frequently

The majority of participants was in favour of this strategy and indicated that it would reduce the barriers to buying healthy foods:

Man, group 1: *'If I saw apples for 60 ct. per kilogram, I would buy 2 kilograms immediately.'*

Woman, group 5: *'Last week, courgettes were on offer; because of that I bought lots of them. Price really is an important factor.'*

Most respondents indicated that they favoured price reductions over quantity discounts (e.g., two for the price of one). An appealing characteristic of discounts is their direct nature (e.g., consumers notice the effect instantly), which is superior to measures of an indirect nature (e.g., insurance premium cutback). Participants did not consider discounts on healthier foods to be patronizing, even if introduced by the government. Still, not all participants could confirm that they would purchase more healthy foods if these were discounted. It was mentioned that other factors, such as knowledge, attitude or ignorance, may still prevent consumers from choosing the healthier options. Yet, most participants emphasized that such discounts would form a good stimulus to purchase healthier foods, especially for those with a low income. Participants suggested that implementation difficulties could be overcome by applying the discounts to organic and seasonal foods.

Man, group 6: *'Everybody knows that organic food is healthier than regular processed foods. However, because it is so expensive very few people purchase this kind of food. If it becomes cheaper, maybe more people would buy it.'*

Some participants stated that discounts will only be effective if they are combined with raising the prices of unhealthier foods:

Man, group 4: *'Discounts make a difference in your budget; you can use this money to buy a bag of crisps.'*

#### IV. Providing a healthy food discount card exclusively for low-income groups

There was an overall enthusiastic response towards the suggestion of providing a healthy food discount card. Both a general discount card, and a card specifically designed for low-income groups received a positive response. Participants indicated that they make heavy use of several discount cards. Since discount cards are very common, participants indicated that an exclusive card for low-income groups would not make them feel stigmatized. An important feature of such a discount card would be that it is valid in all main supermarkets. Some participants stated that the amount of the provided discount should be significant; others disagreed and stated that every little helps:

Woman, group 5: *'I like it very much when I get discounts. Supposing that when you buy a certain amount of fruits and vegetables, you will get a discount of around 10% or 15%, that would help.'*

#### V. Offering small presents, extras, or saving stamps with healthy food items

In general, the subjects responded positively towards this strategy. However, it was suggested that this strategy would work primarily for families with children. It was noticed that children have a large influence on what products are bought and that they are very much tempted by appealing products, mainly snacks. The influence of children in food selection was in particularly named in the focus groups with the Turkish and Moroccan immigrants.

Woman, group 5: *'For example, regular yoghurt comes with a boring wrapping. Children prefer the little deserts provided with a spoon and a sticker. Why can't the regular yoghurt also be made attractive like this?'*

Woman, group 5: *'I think you could apply such a measure to spinach. Children know Popeye and that he gets muscles by eating spinach. However, there is no picture of Popeye on the spinach.'*

#### VI. Making healthy food items cheaper while unhealthy food items more expensive

This strategy was named frequently by the participants throughout the focus groups, even before the interviewers introduced the strategy. Participants stated that making unhealthier foods more expensive would enforce the measure of only discounting healthy foods. Commonly named positive aspects were that this approach steers consumers towards a healthier food selection, but leaves the choice to the consumer. If people wish to eat unhealthy then they pay for it; instead, they can choose cheaper healthier options. Therefore, this intervention was not viewed as being patronizing. Products named that should be made cheaper were: basic products (rice, potatoes, bread); all the foods that are recommended in the nutrition table; wholegrain products; fresh products; fruits and vegetables; meat; and dairy products. Furthermore, the majority of the participants argued that healthier options of comparable products should become cheaper relative to the unhealthier option (e.g., making wholegrain bread cheaper compared to white bread).

#### VII. Subsidizing healthy foods

Most participants considered this to be a fair strategy, as opposed to taxes on unhealthy foods. Subsidies stimulate all consumers to buy higher amounts of healthy foods, while taxes only affect low-income consumers and are regressive in this sense. Similar to discounting, it was argued that a subsidy on healthier foods could reduce the barrier to buying these products. It was also stated that a subsidy is superior to an allowance for low-income groups since a subsidy would apply to everyone and lowers prices directly in the supermarket.

#### VIII. Tax increase on unhealthy food items

In contrast with combining this measure with lowering the prices of healthy foods, only raising the prices of unhealthy foods received mainly negative responses. It was indicated that levy measures are patronizing (in contrast with subsidies) and only effective in creating more tax revenue. Several participants claimed that it would result in opposite effects, and believed that it would make unhealthy foods more attractive (forbidden fruits). Another major issue was the regressive nature of this measure (e.g., it only affects low-income groups). Also, a few of participants stated explicitly that their consumed quantity of unhealthy foods would not decrease as a consequence of higher prices, since owing to the persistent appealing characteristics of these foods they would remain tempting:

Woman, group 4: *'I regularly eat a whole bag of crisps. When I have finished it, I always regret it. I don't think, however, that if the crisps were more expensive it would have prevented me from buying them.'*

#### IX. Premium cutback on health insurance

This measure concerns the allocation of an insurance premium cutback to clients who have a dietary pattern according to dietary guidelines. Participants reacted differently to upon this strategy. The common consensus was that this measure is unworkable and unverifiable.

## Discussion

This study revealed that residents of deprived neighbourhoods view price as a chief factor in food choice. Price was also experienced as a proficient tool to stimulate healthier food choices. Still, consumers indicated that significant effects could only be achieved by combining price with information and promotion techniques. Overall, pricing strategies focusing on encouraging healthy eating were considered to be more constructive than pricing strategies that focused on discouraging unhealthy eating. Highly regarded strategies were: making healthy foods cheaper combined with making unhealthy foods more expensive; providing a healthy food discount card exclusively for low-income groups; and combining price discounts on healthier foods with other marketing techniques such as displaying cheap and healthy foods at the cash desk.

Like previous findings on physical activity, this study showed that finances are a key factor in food selection [29]. Most participants stated they consider the cost when purchasing food; some of the respondents even indicated price to be the most important criterion. Similar results have been reported in previous studies indicating that price, as well as taste and quality, is the most important factor in food selection [16,31] or is the determining factor in buying a certain product or not [32]. This price consideration is important because dietary quality and dietary costs were found to be positively related and because more price-sensitive consumers are less concerned about the health aspects of food [33-36]. Cost conscious consumers may therefore be more inclined to buy unhealthy food alternatives, since those are the lowest cost dietary options [37]. Participants in our study confirmed this statement by arguing that they considered the purchase of vegetables carefully, and that they omit the purchase of organic food owing to its high price.

As price appears to influence food choice, pricing strategies can be a potentially useful tool in steering food choices in a healthy direction. Experiments in controlled settings showed that price reductions are effective in changing food purchases [18]. Studies on fiscal incentives revealed that taxes and subsidies on food may also be effective [19,20,22]. Furthermore, Herman et al. (2006) found that the provision of extra money to low-income consumers to buy fruits and vegetables led to a significant increase in purchases in this category [38]. However, these studies include small, controlled settings and little is known about how consumers would react to larger-scaled pricing strategies. To our knowledge, the recently published Supermarket Healthy Options Project (SHOP) is the first randomized trial on the effect of price incentives on food purchasing behaviour in a real-life setting. This study found significant effects of discounts on the purchase of healthier food items, however no effect was found on nutrient purchases [39]. It remains unclear how the effects of pricing strategies can be enlarged and what contextual variables should be taken into account when implementing such strategies. This focus group study provides additional insight in these issues.

Pricing strategies receiving the most positive responses were: (a) putting healthy foods more frequent on offer; (b) providing discount cards for low-income consumers; (c) making healthy food items cheaper while making unhealthy food items more expensive; and (d) offering little extras with healthy food (in particular when directed at children). When these results are linked to the outcomes of our previously conducted Delphi Study, it can be observed that the experts and the consumers agree on the potential success of making healthy foods cheaper by either discounts or price cuts, as well as offering little extras with healthy foods. In addition to being effective, the experts judged these strategies to be feasible and affordable. Combining price raises of unhealthy foods with price discounts for healthier foods was also viewed to be effective by the expert panel, but was considered to be less feasible and was indicated as not being accepted by the industrial/processing food sector. In contrast with the consumers, the experts did not view the discount card as being effective. The expert panel also rejected a food allowance for low-income groups. The focus groups responded mixed upon this strategy, and did not agree on whether such an allowance would actually lead to the purchase of higher amounts of healthy foods [23].

Next to price, we found that there is a great need for clear-cut information in relation to the healthiness and production processes of food. Healthy products were perceived as being too expensive, but also their genuine healthiness was doubted among the participants. Other authors emphasized similar concerns, stating that price changes will not improve dietary habits because consumers have difficulty in understanding the health effects of food [40], have a poor immediate reflection of prices [41] and do not recognize their diet as being unfavourable [42]. On the other hand, a study comparing the effects of health messages and pricing incentives on food found that pricing alone has a significant effect on choosing healthy foods [43]. Based on our results we suggest that pricing strategies may be most effective when provided with clear nutritional information. A common consensus was that discounts on healthy foods should be supported by widespread commercials and advertisements which inform the consumer about the healthiness of the product along with the fact that it is on offer. This strategy can be combined with marketing strategies making the healthier food more appealing and attractive (e.g., shelf placement, packaging). Eikenberry et al., reported related conclusions in a study on perceptions and motivations for healthy eating and stated that: "focusing on quick and easy, healthy, less expensive food preparation or selection of more convenient yet inexpensive food may help overcome barriers" [44].

To our knowledge, this is the first study that gives an insight into the perception of residents of deprived neighbourhoods with regard to food pricing strategies. Due to the use of the focus group technique we were not only able to study if pricing strategies may be effective, but also how they may be most effective. The results are a good starting point for future price intervention studies. Still, some limitations of this study must be noted. Since pricing strategies were the main interest in this study, more focus was put on this than on other interventions. This may have led to an over-estimation of the perceived potential effectiveness. In addition, the discussed strategies were formulated by the researchers, and were not selected by the participants themselves. However, we made an effort to propose the strategies in a neutral manner, without being suggestive. Also, respondents came up with pricing strategies in answering an open-ended question, and indicated price as being important without it being suggested to them. Secondly, we believe that the use of the nine pricing strategies that resulted from a previously conducted Delphi study is a merit of our study. Those nine strategies represented approaches that were viewed as potentially successful by a group of carefully selected experts and had already been tested on perceived feasibility, effectiveness and affordability issues [23]. Proposing these strategies to the target population enabled us to gain a unique insight into which strategies are evaluated positively by both experts and consumers. Thirdly, it must be mentioned that we included residents of deprived neighbourhoods, which can not be regarded equal to residents with a low SES. Of the total sample (n = 59), 22 participants were low educated; 18 were unemployed, and 26 had an income below standard. A final limitation is that no 100% data saturation was achieved at the end of this study. One or two additional focus groups may have led to full data saturation. However, we specifically sampled different cultural backgrounds and in two different cities to ensure a large variation in data. In this setting a data saturation of 96% can be viewed as satisfactory.

## Conclusion

Marketing research indicated price as one of the most important tools to influence consumer behaviour [7,9], and small-scale experiments showed that pricing strategies may be useful in changing dietary behaviour [45,46]. Still, little is known about how consumers would react to larger-scaled pricing strategies. This study found that consumers feel that price could be an efficient technique to stimulate healthier food choices, especially when different techniques are combined. This includes a combination of: 1) raising the prices of unhealthier foods with lowering prices of healthier foods; 2) price discounts on healthier foods with clear nutritional information; and 3) price discounts on healthier foods with other marketing techniques, such as displaying cheap and healthy foods at the cash desk. Hence, large-scale experimental studies are warranted on the effects of such pricing strategies on purchasing behaviour. These studies should focus on larger purchasing settings such as supermarkets, include price elasticity issues, study the effect of additional nutritional information, as well as the effects of signing the discounted products in different ways (e.g., this product is on sale or is the healthier and cheaper choice).

## Appendix 1 - Attitudes and perceptions towards food prices, main results

*Food selection is based on cutting down expenses, not on preferences

*Organic foods, fruits, vegetables, higher quality food, dairy products, meat, and healthier food options of comparable food items are too expensive

*Price is a determining factor

*A low income restricts food options, especially for fruit, vegetables, meat and organic foods

*There are little opportunities for sufficient dietary variation on a small budget

*Convenience food is cheaper than fruit and vegetables, this creates an imbalance

*Solutions are to buy seasonal foods, frozen fruit and vegetables, go to the weekly market or to cheaper supermarkets

*It is not merely the price, there is a need for clear nutritional information

*Other important factors in food choice are convenience, attractiveness, calorific content, and taste

*Along with attractive prices, convenience foods are often also more appealing owing to fancy packaging and small free gifts

## Competing interests

The authors declare that they have no competing interests.

## Authors' contributions

WEW: design; acquisition of data; analysis and interpretation of data; drafting the manuscript. AM: analysis and interpretation of data, critically revision of the manuscript for important intellectual content. AJS: critically revision of the manuscript for important intellectual content. JCS: critically revision of the manuscript for important intellectual content. IHMS: conception and design; critically revision of the manuscript for important intellectual content. All authors read and approved the final manuscript.
